# What should we report? Lessons learnt from the development and implementation of serious adverse event reporting procedures in non-pharmacological trials in palliative care

**DOI:** 10.1186/s12904-021-00714-5

**Published:** 2021-01-20

**Authors:** Lesley Dunleavy, Danni Collingridge Moore, Ida Korfage, Sheila Payne, Catherine Walshe, Nancy Preston

**Affiliations:** 1 International Observatory on End of Life Care, Faculty of Health and Medicine, Division of Health Research, Health Innovation One, Sir John Fisher Drive, Lancaster University, Lancaster, LA1 4AT UK; 2 Department of Public Health, Erasmus MC, Rotterdam, Netherlands

**Keywords:** Clinical trial, Palliative care, Serious adverse event, Dementia, Cancer

## Abstract

**Background/aims:**

Serious adverse event reporting guidelines have largely been developed for pharmaceutical trials. There is evidence that serious adverse events, such as psychological distress, can also occur in non-pharmaceutical trials. Managing serious adverse event reporting and monitoring in palliative care non-pharmaceutical trials can be particularly challenging. This is because patients living with advanced malignant or non-malignant disease have a high risk of hospitalisation and/or death as a result of progression of their disease rather than due to the trial intervention or procedures. This paper presents a number of recommendations for managing serious adverse event reporting that are drawn from two palliative care non-pharmacological trials.

**Methods:**

The recommendations were iteratively developed across a number of exemplar trials. This included examining national and international safety reporting guidance, reviewing serious adverse event reporting procedures from other pharmacological and non-pharmacological trials, a review of the literature and collaboration between the ACTION study team and Data Safety Monitoring Committee. These two groups included expertise in oncology, palliative care, statistics and medical ethics and this collaboration led to the development of serious adverse event reporting procedures.

**Results:**

The recommendations included; allowing adequate time at the study planning stage to develop serious adverse event reporting procedures, especially in multi-national studies or research naïve settings; reviewing the level of trial oversight required; defining what a serious adverse event is in your trial based on your study population; development and implementation of standard operating procedures and training; refining the reporting procedures during the trial if necessary and publishing serious adverse events in findings papers.

**Conclusions:**

There is a need for researchers to share their experiences of managing this challenging aspect of trial conduct. This will ensure that the processes for managing serious adverse event reporting are continually refined and improved so optimising patient safety.

**Trial registration:**

ACTION trial registration number: ISRCTN63110516 (date of registration 03/10/2014).

Namaste trial registration number: ISRCTN14948133 (date of registration 04/10/2017).

**Supplementary Information:**

The online version contains supplementary material available at 10.1186/s12904-021-00714-5.

## Background

More research is needed in palliative care to improve the evidence base that underpins clinical practice [1], especially as the need for palliative care is predicted to increase substantially by 2060 [2]. There is a commensurate need to increase the number of high quality trials in palliative care as they are an optimal design for testing the effectiveness of treatments and therapeutic interventions [3, 4]. Many interventions and treatments commonly used in palliative care have little supporting trial evidence [5]. Clinical trials, as well as testing effectiveness, also need to assess whether the novel treatment or intervention is in fact safe [6].

Safety reporting procedures aim to capture any adverse events that may arise during a trial [7]. Trial protocols should contain details of how adverse events are to be identified, collected, assessed, reported and managed [8]. Findings papers should also report on the adverse events that have occurred during a trial [7, 9, 10]. There are internationally agreed definitions and reporting procedures for pharmacological trials [11]. In a clinical trial, an adverse event is any untoward medical occurrence that is experienced by a trial participant which is not necessarily related to the intervention [12]. The adverse event is classified as serious when, at any dose, it results in: death, is life-threatening, requires inpatient hospitalisation or prolongation of existing hospitalisation, results in persistent or significant disability/incapacity or is a congenital anomaly/birth defect [12].

Monitoring of adverse events during a trial is key to ensuring patient safety but structures and processes, including nomenclature, can vary depending on the funder, trial type and jurisdiction [13]. Generally, an internal study team or group is responsible for the day to day running of the trial while a Trial Steering Committee, made up of largely independent members including patient representation, provides additional scrutiny [13]. An independent Data Safety Monitoring Committee may also be set up, more commonly in pharmaceutical trials, to monitor un-blinded safety and efficacy data and if required recommend the trial is stopped to safeguard the interests of participants [13, 14]. Ethical approval processes can vary internationally [15] but a research ethics committee’s role is to review the potential risks of a study [11]. Requirements for reporting adverse events to research ethics committees can vary between nations [16] but international guidance recommends that unexpected serious adverse events related to the intervention (SUSARs) be promptly reported [11].

There is evidence that serious adverse events, such as psychological distress, can occur in non-pharmacological trials [17]. This paper focuses on serious adverse event reporting in palliative care non-pharmaceutical trials as there is a lack of guidance for researchers. This is also an issue outside palliative care. One review of psychological trials highlighted an over reliance on the definition used in pharmacological trials and that researchers did not identify which serious adverse events might likely arise from a specific intervention in a particular population [18].

The definition of a palliative care population can vary [19, 20] but in this paper a palliative care trial focuses on those patients living with advanced malignant or non-malignant disease and their family carers. This group of patients are viewed as vulnerable as they have complex physical, psychosocial and spiritual needs and can have a limited life expectancy [21]. They are cared for in diverse clinical settings and receive care from specialist and/or generalist palliative care professionals. Non-pharmacological palliative care interventions are heterogeneous. Typically, they are complex interventions that reflect a holistic and multi-disciplinary approach to care [22] with quality of life and/or symptom control being the primary outcome [22–24] rather than survival or disease response [25]. Interventions may be taken from other patient populations and applied to those living with advanced disease [26] or developed specifically to meet the needs of this patient group [27]. The characteristics of a non-pharmacological palliative care trial make implementing serious adverse event reporting procedures challenging.

The challenges of applying the standard serious adverse event definitions and reporting procedures was considered in two recent palliative care non-pharmacological trials. The ACTION study was a cluster randomised controlled trial assessing the effects of an advance care planning programme on the quality of life of patients with advanced lung or colorectal cancer. The trial took place in six European countries and recruited 1117 participants in the hospital setting [28]. The Namaste Care study was a feasibility cluster randomised controlled trial. The trial took place in nursing homes in the UK and focused on the psychosocial Namaste Care intervention for residents living with advanced dementia [29]. Research Ethics Committee approval was obtained in all six countries taking part in the ACTION study (NRES Committee North West - Liverpool East 14/NW/1189) and in the UK for the Namaste study (Wales Research Ethics Committee 5 Bangor 17/WA/0378). Written informed consent was obtained for all participants with consent being provided by a proxy in the Namaste trial as residents lacked capacity.

Given the health status of participants in the ACTION trial and the Namaste trial, there was a relatively high risk of death and/or hospitalisation for participants during the study. These events were not anticipated to be related to the receipt of the intervention or the trial procedures and similar issues have been raised in critical care trials [30]. A challenge in both studies was how to ensure serious adverse events related to the trial intervention or procedures were recorded while preventing unnecessary and burdensome reporting processes for both study coordinating centre staff and clinicians. There was a risk that reporting all serious adverse events would result in those potentially related to the intervention being missed [31]. There was also a risk that clinical staff would not report serious adverse events because they were not pharmaceutical trials.

This paper outlines a number of recommendations (see Table 1) that were drawn from the learning from these two exemplar trials. The recommendations may be useful for others who are developing and implementing serious adverse event reporting procedures in palliative care non-pharmaceutical trials.

**Table 1:** Recommendations for managing serious adverse event reporting procedures in palliative care non-pharmacological trials

**Fig. 1:**
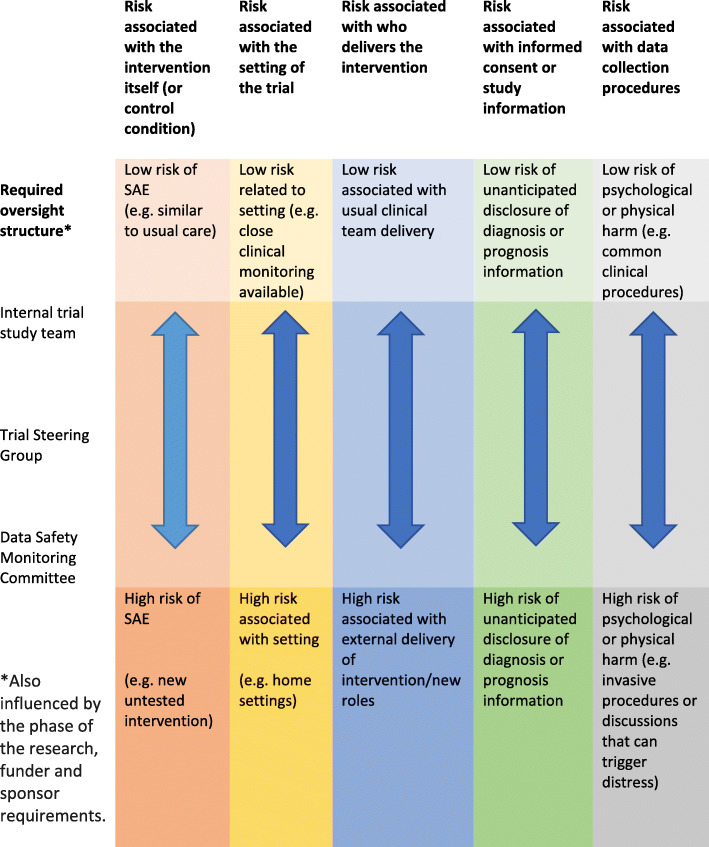
Potential structure for trial oversight in palliative care non-pharmacological trials

• Factor in adequate time at the study planning stage to develop serious adverse event reporting procedures especially in a multi-national study or for research naïve settings such as a nursing home. • Review level of trial oversight required (see Fig. 1) • Define what a serious adverse event is in your trial, based on your study population, including their health state, the expected risks and the type of events that should be reported. • Develop documentation to support serious adverse event reporting. • Implement serious adverse event reporting procedures. • Monitor serious adverse events during the trial. • Refine the reporting procedures during the trial if necessary. • Report the serious adverse events that occur during the trial in the final report papers.	

## Methods

A number of strategies were used to develop the serious adverse event reporting procedures for the ACTION trial. Initially, the procedures of other pharmacological and non-pharmacological trials were reviewed for guidance. This was in addition to the national and international guidance available to guide serious adverse event reporting in clinical trials [7, 8, 10, 11, 32]. This formed the basis of the serious adverse event form used in the study. A Data Safety Monitoring Committee was set up, as this was a requirement in the UK, an approach then approved by all trial consortium members. The Data Safety Monitoring Committee recommended a proactive rigorous approach to the monitoring of serious adverse events during the trial (see Table 3 for further details). Development of the serious adverse event reporting procedures was a collaborative process between the ACTION trial consortium and the trial’s Data Safety Monitoring Committee. Both groups comprised a diverse group of clinical and academic professionals from across Europe and included expertise in oncology, palliative care clinical practice and research, including trials, statistics, as well as medical ethicists. This collaboration led to the definition of a serious adverse event in this study (see Table 2).

**Table 2:** Defining what a serious adverse event is in your trial

	ACTION trial	Namaste trial
**What is the study population?**	Advanced colorectal or lung cancer patients with an approximate 50% one-year survival rate. It would not be unexpected that patients may die or be admitted to hospital while taking part in the trial.	Nursing home residents living with advanced dementia (FAST score 6 or 7). In a previous study evaluating the Namaste Care programme, early deaths (< 2 months) were not uncommon in the advanced dementia population [33].
**What are the expected risks?**	Patient and/or carer distress due to the intervention and/or completion of questionnaires. The risks were expected to be limited in those countries where advance care planning conversations are considered to be part of routine care and mostly validated questionnaires were being used in the study.	The anticipated risks for residents of taking part were viewed as low as the core elements of the programme are sensory activities that involve music, massage, colour, taste and scents. These core elements are viewed as best practice in dementia and end of life care. A potential risk identified was a skin reaction to Namaste Care activities e.g. massage oils or the actigraphy watch that was being used for data collection, with anaphylaxis being viewed as a potential serious adverse event. Nursing home staff completed proxy questionnaires on behalf of the residents taking part in the study as they lacked capacity.
**What events should be reported?**	‘We ask you to complete this form for every event in the study that takes a course that is significantly more unfavourable to study participants than foreseen in the normal course of the illness.’ *All hospital stays of at least one night and deaths in both arms of the trial were included in reports for the Data Safety Monitoring Committee.	Only deaths, hospitalisations, life threatening or medically significant/important events related to the intervention or data collection procedures were to be reported as serious adverse events.

During the trial, a review of the literature was carried out to explore how the serious adverse event reporting procedures of the ACTION study compared with other trials of palliative care psychological interventions (see Additional file 1). The review highlighted that there is a lack of evidence of how serious adverse events should be monitored in these type of studies. How the study teams planned to manage psychological distress and deal with concerns raised from questionnaire responses were sometimes reported in the published trial protocols. There was also a lack of reporting of serious adverse events in the final reports of included studies which could suggest that no serious adverse events have occurred, they were not recognised or recorded or they were recorded but not reported [18].

The recommendations outlined below were iteratively developed from the learning across both trials.

## The recommendations

Experience from both trials highlighted the need to factor in adequate time at the study planning stage to develop serious adverse event reporting procedures that reflected the study population, the intervention being tested and that aligned with international, national and local procedures. The Namaste trial also required additional time as the nursing home sites had not taken part in a previous trial and for some of the homes, this was their first experience of research.

### Defining what a serious adverse event is in your trial

The importance of defining what a serious adverse event is in your trial based on your study population was identified. This definition should take account of their health status, the expected risks and the type of events that should be reported. How this process was operationalised in the two trials is described in Table 2. In the Namaste trial, patient and public involvement representatives provided advice on the wording of participation information [34] and questionnaires to try and reduce the risk of distress.

### Documentation to support serious adverse event reporting

Serious adverse event standard operating procedures and reporting forms were developed for both trials. The Clinical Trial Unit that was managing the Namaste trial data had limited experience of supporting non-pharmaceutical trials. Their standard reporting procedures had to be adapted to fit the trial design and clinical setting which added additional time to the study set up process. In the ACTION trial, a form for documenting routine hospital admissions was produced that asked for reason and length of admission. In both trials, a form was created to document all deaths which included the date and cause of death, in the ACTION trial place of death was also documented.

### Implementation of serious adverse event reporting procedures

In the ACTION trial, oncologists and research nurses were experienced in pharmacological trial serious adverse event reporting procedures but less so in non-pharmacological studies. Informal training was provided at the start of study and support was available throughout the trial and if a serious adverse event was suspected. In the Namaste trial, nursing home staff were unsurprisingly largely research naïve so a research manual was developed to explain reporting procedures to non-research staff. Formal research training was provided at the start of the study and support was available throughout the trial and if a serious adverse event was suspected.

### Monitoring of serious adverse events during the trial

Multiple strategies were used to monitor serious adverse events in both trials and reporting procedures were refined during the trial as necessary (see Table 3). As recommended by the Consort guidelines [7], both passive and active surveillance strategies were used. Passive surveillance involved the recording of spontaneously reported serious adverse events by patients, their proxies or health care professionals. In the ACTION trial, active surveillance involved the review, by the Data Safety Monitoring Committee, of the total number of patients screened for eligibility, who was eligible, asked for consent, and included plus response rates per study arm and per tumour type, the primary outcome measure and hospital admission and death data in both arms of the trial. In the Namaste trial, a review of baseline questionnaires highlighted the need to monitor patient pain scores and guidance for highlighting concerns to the nursing home manager was developed.

**Table 3:** Monitoring of serious adverse events during the trial

ACTION trial	Namaste trial
Regular telephone and face to face contact with clinical sites. Data Safety Monitoring Committee review of: • Serious adverse event forms • Items from the Quality of Life questionnaires related to distress • Routine hospital admission and expected death information • Total number of patients screened for eligibility, who were eligible, asked for consent, and included plus response rates per study arm and per tumour type Liaison with clinical staff, as necessary, to ensure an appropriate plan of care was put into place.	Regular telephone and face to face contact with nursing home sites. Review of serious adverse event forms by study coordinating centre and findings reported to the Trial Steering Committee. Monitoring of pain scores reported during the trial. Study coordinating staff to report concerns to nursing home manager if pain scores high.

### Reporting of the serious adverse events that occur during the trial

The serious adverse events that occurred were reported in the final report papers. In the ACTION trial, three serious adverse events related to the intervention were reported; one patient became distressed after reading the study information materials and two after having participated in the advance care planning conversations. They were resolved through conversations with the patients [35]. In the Namaste trial, there were no serious adverse events reported but one adverse event arose from use of the actigraph device used for data collection. Bruising was observed on one individual, with no lasting effect [36].

## Discussion

The need to improve the quality of reporting of serious adverse events in trials has been recognised [7, 9] but there is a lack of practical guidance on how to manage this process, particularly in palliative care non-pharmacological trials. This may be because published trial protocols and results papers may have limited space to document these processes and/or they are challenging to implement because of the characteristics of a palliative care trial. This paper addresses this issue by presenting a number of recommendations based on the lessons learnt from managing serious adverse event reporting procedures in two non-pharmacological trials in palliative care.

When designing a palliative care non-pharmaceutical trial the possibility that serious adverse events may occur should be not be dismissed and should be actively considered, including ‘worst case scenarios’. In pharmaceutical trials, the potential for serious adverse events to occur is evaluated in four phases of trial development. Phase I trials, historically referred to as ‘toxicity trials’, test a new drug in a small number of participants to identify the dose range and the drug’s safety profile [16, 37]. Phase II trials evaluate safety in a larger group of participants and set the dosage schedule for further phases. Phase III trials are usually double blind randomised controlled trials involving more participants and they assess efficacy and serious adverse events between intervention and control arms. Phase IV studies are post marketing studies and evaluate serious adverse events related to longer term use [16, 38]. The four stages of the Medical Research Council framework for developing complex interventions reflect the phases of drug development [39, 40]. As discussed previously, palliative care non-pharmaceutical trials typically involve complex interventions. The potential for serious adverse events to occur is something that should be explicitly explored earlier in their development and conduct. For example, in the feasibility/piloting stage, one of the trial’s objectives should be to determine the type and consequences of any serious adverse events related to the intervention or study procedures prior to a definitive trial [41]. Reviews of feasibility/pilot studies, however, show that this is not always the case [42, 43].

This paper also contributes to the discussion regarding trial safety oversight in the context of palliative care non-pharmaceutical trials. Setting up a Data Safety Monitoring Committee or Trial Steering Committee with appropriate expertise can be time consuming, an issue also raised in the general trial literature [44]. This can be more challenging for international studies when there may be a number of different local regulatory requirements to incorporate into the process. The criteria for determining the need for a Data Safety Monitoring Committee are not well defined, even in pharmaceutical trials [44]. Research ethics committees, as in pharmaceutical trials, should review whether potential serious adverse events have been considered and how they are going to be monitored in these type of studies [11].

The MORECare recommendations for evaluating complex interventions in end of life care do not cover serious adverse event reporting or how safety should be monitored in this context, including the role of ethics committees and other monitoring committees [5]. This is an area of palliative care trial methodology that requires further research. In this context, a risk assessment matrix may help researchers determine the type of oversight committee required for their trial (see Fig. 1) but this requires further research. In the palliative care context, risks associated with introducing the trial may also need to be considered, as this will be dependent on the patient’s level of awareness and the communication skills of the recruiter [45].

## Conclusions

There may be a greater level of risk associated with pharmaceutical trials but as our experience has highlighted non-pharmaceutical trials are not, as is sometimes assumed, risk free. There is a need for those involved in non-pharmaceutical trials to share their experiences of managing this challenging aspect of trial conduct. This will ensure the procedures for managing serious adverse events are continually refined and improved so optimising patient safety, with further research warranted.

## Supplementary Information

**Additional file 1.**

## Data Availability

All data generated or analysed during this study are included in this published article and its supplementary information files. In addition, the ACTION and Namaste trial protocol and findings papers are open access.

## References

[1] Visser C, Hadley G, Wee B (2015). Reality of evidence-based practice in palliative care. Cancer Biol Med.

[2] Sleeman KE, de Brito M, Etkind S (2019). The escalating global burden of serious health-related suffering: projections to 2060 by world regions, age groups, and health conditions. Lancet Glob Health.

[3] Walshe C (2017). Palliative care research: state of play and journal direction. Palliat Med.

[4] Bouça-Machado R, Rosário M, Alarcão J (2017). Clinical trials in palliative care: a systematic review of their methodological characteristics and of the quality of their reporting. BMC Palliat Care.

[5] Higginson IJ, Evans CJ, Grande G (2013). Evaluating complex interventions in end of life care: the MORECare statement on good practice generated by a synthesis of transparent expert consultations and systematic reviews. BMC Med.

[6] Hunsinger MM, Smith PS, Rothstein HD (2014). Adverse event reporting in nonpharmacologic, noninterventional pain clinical trials: ACTTION systematic review. Pain.

[7] Ioannidis JPA, Evans SJW, Gøtzsche PC (2004). Better reporting of harms in randomized trials: an extension of the CONSORT statement. Ann Intern Med.

[8] Chan A-W, Tetzlaff JM, Gøtzsche PC (2013). SPIRIT 2013 explanation and elaboration: guidance for protocols of clinical trials. British Med J.

[9] Montgomery P, Grant S, Mayo-Wilson E (2018). Reporting randomised trials of social and psychological interventions: the CONSORT-SPI 2018 extension. Trials.

[10] Schulz KF, Altman DG, Moher D (2010). CONSORT 2010 statement: updated guidelines for reporting parallel group randomised trials. BMJ (Clinical research ed).

[11] European Medicines Agency (2016). ICH E6 (R2) good clinical practice.

[12] Good Clinical Practice Network (2019). Glossary.

[13] Lane JA, Gamble C, Cragg WJ (2020). A third trial oversight committee: functions, benefits and issues. Clin Trial.

[14] DeMets DL, Ellenberg SS (2016). Data monitoring committees—expect the unexpected. N Engl J Med.

[15] Preston N, van Delden JJ, Ingravallo F (2020). Ethical and research governance approval across Europe: experiences from three European palliative care studies. Palliat Med.

[16] Wallace S, Myles PS, Zeps N (2016). Serious adverse event reporting in investigator-initiated clinical trials. Med J Aust.

[17] Day F, McMurran M, Duley L (2013). The process of stopping recruitment and trial treatment in a trial of a psychological therapy for people with personality disorder following a safety alert. Trials.

[18] Duggan C, Parry G, McMurran M (2014). The recording of adverse events from psychological treatments in clinical trials: evidence from a review of NIHR-funded trials. Trials.

[19] Radbruch L, De Lima L, Knaul F (2020). Redefining palliative care–a new consensus-based definition. J Pain Symptom Manag.

[20] Sepúlveda C, Marlin A, Yoshida T (2002). Palliative care: the World Health Organization's global perspective. J Pain Symptom Manag.

[21] Verkissen MN, Hjermstad MJ, Van Belle S (2019). Quality of life and symptom intensity over time in people with cancer receiving palliative care: results from the international European palliative care cancer symptom study. PLoS One.

[22] Van Mechelen W, Aertgeerts B, De Ceulaer K (2013). Defining the palliative care patient: a systematic review. Palliat Med.

[23] Gaertner J, Siemens W, Daveson BA (2016). Of apples and oranges: lessons learned from the preparation of research protocols for systematic reviews exploring the effectiveness of specialist palliative care. BMC Palliat Care.

[24] Vinches M, Neven A, Fenwarth L (2020). Clinical research in cancer palliative care: a metaresearch analysis. BMJ Support Palliat Care.

[25] Hui D, Parsons HA, Damani S (2011). Quantity, design, and scope of the palliative oncology literature. Oncologist.

[26] Warth M, Koehler F, Aguilar-Raab C (2020). Stress-reducing effects of a brief mindfulness intervention in palliative care: results from a randomised, crossover study. Eur J Cancer Care.

[27] Bakitas MA, Dionne-Odom JN, Ejem DB (2020). Effect of an early palliative care Telehealth intervention vs usual care on patients with heart failure: the ENABLE CHF-PC randomized clinical trial. JAMA Intern Med.

[28] Rietjens JAC, Korfage IJ, Dunleavy L (2016). Advance care planning - a multi-Centre cluster randomised clinical trial: the research protocol of the ACTION study. BMC Cancer.

[29] Froggatt K, Patel S, Perez Algorta G (2018). Namaste care in nursing care homes for people with advanced dementia: protocol for a feasibility randomised controlled trial. BMJ Open.

[30] Moskowitz A, Andersen LW, Holmberg MJ (2020). Identification, collection, and reporting of harms among non-industry-sponsored randomized clinical trials of pharmacologic interventions in the critically ill population: a systematic review. Crit Care.

[31] Hardy J, Shelby-James T, Currow DC (2010). Research in palliative care: is death always an adverse event?. Intern Med J.

[32] Health Research Authority (2019). Safety reporting.

[33] Stacpoole M, Hockley J, Thompsell A (2017). Implementing the Namaste care program for residents with advanced dementia: exploring the perceptions of families and staff in UK care homes. Ann Palliat Med.

[34] Crocker JC, Ricci-Cabello I, Parker A (2018). Impact of patient and public involvement on enrolment and retention in clinical trials: systematic review and meta-analysis. BMJ (Clinical research ed).

[35] Korfage IJCG, Arnfeldt Christensen CM, Billekens P, Bramley L, Briggs L (2020). Advance care planning in patients with advanced cancer: a 6-country, cluster-randomised clinical trial. PLoS Med.

[36] Froggatt K, Best A, Bunn F (2020). A group intervention to improve quality of life for people with advanced dementia living in care homes: the Namaste feasibility cluster RCT. Health Technol Assess (Winchester, England).

[37] Adashek JJ, LoRusso PM, Hong DS (2019). Phase I trials as valid therapeutic options for patients with cancer. Nat Rev Clin Oncol.

[38] Friedman LM, Furberg C, DeMets DL (2010). Fundamentals of clinical trials. 4th ed.: springer.

[39] Campbell M, Fitzpatrick R, Haines A (2000). Framework for design and evaluation of complex interventions to improve health. BMJ (Clinical research ed).

[40] Craig P, Dieppe P, Macintyre S (2013). Developing and evaluating complex interventions: the new Medical Research Council guidance. Int J Nurs Stud.

[41] Walshe C, Roberts D, Calman L (2020). Peer support to maintain psychological wellbeing in people with advanced cancer: findings from a feasibility study for a randomised controlled trial. BMC Palliat Care.

[42] Blatch-Jones AJ, Pek W, Kirkpatrick E (2018). Role of feasibility and pilot studies in randomised controlled trials: a cross-sectional study. BMJ Open.

[43] Morgan B, Hejdenberg J, Hinrichs-Krapels S (2018). Do feasibility studies contribute to, or avoid, waste in research?. PLoS One.

[44] Calis KA, Archdeacon P, Bain R (2017). Recommendations for data monitoring committees from the clinical trials transformation initiative. Clinical Trials.

[45] Holm M, Alvariza A, Fürst C-J (2017). Recruiting participants to a randomized controlled trial testing an intervention in palliative cancer care – the perspectives of health care professionals. Eur J Oncol Nurs.

