# Supplementary material for: What should we report? Lessons learnt from the development and implementation of serious adverse event reporting procedures in non-pharmacological trials in palliative care
Source: BMC Palliat Care. 2021 Jan 20;20:19. doi: 10.1186/s12904-021-00714-5 (PMC7819235; doi:10.1186/s12904-021-00714-5)
Supplement: Supplementary file 1 — Additional file 1. [file 12904_2021_714_MOESM1_ESM.docx]

**Additional file 1**

A review of the literature was carried out by LD and DCM to explore how serious adverse event reporting procedures of the ACTION study compared with other palliative care randomised controlled trials of psychological interventions. The review focused on palliative care randomised controlled trials published protocols and final reports. Three databases were searched (Medline, Embase and PsychINFO) from April 2010 to April 2016 and data were extracted from 17 articles meeting predefined eligibility criteria.

**Table 1 Example search strategy**

| **Medline**  palliat*  hospice*  terminal care  terminal care mesh no exp  palliative care/medicine mesh  randomi?ed controlled trial*  randomised controlled trial mesh pub + topic  **limits:** human, from 04/10 to 04/16, RCT |
| --- |

**Table 2 Inclusion/Exclusion criteria**

| Inclusion | Exclusion |
| --- | --- |
| Types of papers  Published protocols of palliative care RCTs focusing on psychological interventions aimed at patients and/or family carers.  Published papers outlining the results of psychological palliative care interventional RCTs aimed at patients and/or family carers.  Pilot/feasibility studies as well as full scale RCTs will be included.  These papers will be reviewed for information on the reporting of serious adverse events and how serious adverse events were to be or were monitored. | Types of papers  Conference abstracts/papers and systematic reviews  Non randomised trials  Those studies including patients with both curable and incurable disease if it is impossible to distinguish findings between groups.  Pharmacological/complementary therapy RCTs |
| Study Population  Cancer   - Adult cancer patients with incurable disease (defined by tumour staging) - Non-professional carers of cancer patients with incurable disease   Non-Cancer   - Adults with a progressive, life threatening disease (defined by classifications of disease severity such as New York Heart Association Class NB This would include patients classed in the literature as ‘frail elderly’   if they were receiving an intervention that was clearly a palliative care psychological intervention.)   - Non-professional carers of patients with a progressive, life threatening disease | Study Population  Adult cancer patients with potentially curable disease  Care of chronic non-life threatening conditions without a curative treatment option. |
| Type of trial  Studies that focus primarily on meeting the psychological/emotional needs of patients and/or their family caregivers including psychoeducational interventions such as advance care planning. |  |

Records identified through database searching
(n = 1,275)

Records after duplicates removed
(n = 1,080)

Records screened
(n = 1,080)

Records excluded
(n = 1,012)

Full-text articles excluded n = 51
(n = 11 not palliative care,
n = 40 no information)

Studies included in the synthesis
(n = 17)

Full-text articles assessed for eligibility
(n = 68)

**Figure 1 Prisma Flowchart**

**Table 3: Data extraction table**

| **Author/type of paper/country of origin** | **Study aim** | **Setting/Sample** | **Type of Intervention** | **Monitoring committee** | **Serious adverse event reporting/data** | **Monitoring of questionnaire data** | **Management of distress** |
| --- | --- | --- | --- | --- | --- | --- | --- |
| Bernacki et al^1^  2015  Protocol paper  US | To describe the protocol of a multi component structured communication intervention that aims to assess receipt of goal concordant care and peacefulness at the end of life. | Cancer centre, patients with advanced cancer, life expectancy under 12 months with their surrogate | multi component structured communication | No information | An approved protocol to monitor for adverse events is followed. | Monitor responses to PHQ-9 (depression) and GAD-7 (anxiety/pain) scales and inform clinical team if concerns | Additional support for patients with symptoms of psychological distress is available |
| Bove et al^2^  2015  Protocol paper  Denmark | To test the efficacy of a minimal home based psycho-educative intervention versus usual care for managing anxiety and dyspnoea in patients with severe COPD | patients home  severe COPD, GOLD C or D | manual based on CBT, psychoeducation and standard care versus standard care | No information | To the best of our knowledge there is no documentation of the risks associated with participating in CBT or psycho-educative interventions. The intervention is perceived as unharmful and should not have adverse effects. | No information | During the intervention or follow up, the PI will encourage the participants to seek help from their GP or in the pulmonary clinic if there is a need for additional professional support. |
| Houben et al^3^  2014  Protocol paper  the Netherlands | To test the efficacy of structured advance care planning on quality of end of life communication and quality of end of life care. | severe to very severe COPD (GOLD III-IV), discharged after hospital admission for a COPD exacerbation and family carer | single structured advance care planning session with a respiratory nurse versus usual care | The study will be monitored according to guidelines of the Dutch Federation of University medical centres | We hypothesise that structured advance care planning will not result in increased symptoms of anxiety and depression. | No information | No information |
| Kimmel at el^4^  2015  Protocol paper  US | To test the efficacy of family centred advance care planning on congruence in treatment preferences, health care utilization and QOL | Out patients  advanced HIV/AIDS with comorbidities | family centred facilitated advance care planning versus health living control | Reviewed twice a year by the trial Safety Monitoring Committee | No information | No information | No information |
| Kruizinga et al^5^  2013  Protocol paper  the Netherlands | To examine whether an assisted reflection on life events and ultimate life goals can improve the QOL of cancer patients. | Hospital  advanced cancer, exclusion psychiatric disease | Two consultations with a spiritual counsellor using an interview scheme supported by e application versus usual care | No information | Expect to find positive outcome, realise negative experiences may also be induced, may become anxious and depressed when they bring life events from the past back into memories. We will measure anxiety and depression. | No information | No information |
| Lo et al^6^  2015  Protocol paper  Canada | To test the efficacy of a semi structured manualised psychotherapeutic intervention to alleviate distress and promote well-being. | Oncology outpatients advanced cancer or metastatic cancer | Managing cancer and living meaningfully (CALM)) versus usual care | No information | No information | No information | Intervention arm: if therapist considers patient to be at acute risk of suicide/ significant worsening of depression or other psychiatric comorbidity that require treatment will be referred for psychiatric assessment and treatment Usual care: participant reports suicidal intent the PIs are contacted and the patent is assessed by a psychiatrist. |
| Reid et al^7^  2014  Protocol paper  UK | To develop and evaluate a psychoeducational intervention aimed at improving the psychological well- being of advanced cancer patients with cachexia and their lay carers | Hospice  advanced cancer patients with cachexia and their lay carers | psychoeducational DVD versus usual care (offered DVD after the trial) | The overall conduct of the study will be monitored by a UK CRC CTU. | No information | No information | Content of DVD could potentially be upsetting, follow up telephone call after receive DVD, advised to contact GP for additional support |
| Scheffold et al^8^  2015  Protocol  Germany | To examine the efficacy of a brief, manualized individual psychotherapy for patients with advanced cancer: Managing Cancer and Living Meaningfully (CALM) | Psycho-oncology outpatients  advanced cancer, highly distressed | Managing Cancer and Living Meaningfully (CALM) | No information | Research assistant evaluates exclusion criteria: (c) acute suicidality (concrete suicidal thoughts and/or plans, in which case psychiatric care is provided immediately). At the point of exclusion, study results up to this date and reasons for exclusion will be documented. These data will be further analyzed in the drop-out analysis. | No information | If exclusion criteria occur during the intervention psychiatric care will be provided immediately. |
| Walczak et al^9^  2014  Protocol paper  Australia | To describe the protocol of a nurse led communication support programme to facilitate patients and caregivers efforts to communicate EOLC issues with their health care team. | medical oncology clinics  advanced cancer, prognosis less than 12 months and care giver if possible | No information | No information | Accepted adverse event monitoring procedures will be followed. | No information | Appropriate referral to psychological services at participating treating hospitals will be made as required |
| Chochinov et al^10^  2011  Results paper  Canada, US and Australia | To investigate whether dignity therapy is better than standard palliative care or client centred care in terms of reducing psychosocial, existential and spiritual distress in patients who are terminally ill. | hospital or community (hospice or home)  life expectancy 6 months or less, cancer or non- cancer patients | dignity therapy or client centred care or standard palliative care | No information | Discussion: not aware of any instance in which patients withdrew from dignity therapy because of feeling upset or distressed. The only safety issue, concerned a few occasions where family members were dissatisfied with the generativity document. In those instances, they felt the patient had become too ill to give fulsome responses, or that the answers provided a distorted image of the participant. Patients who are delirious or otherwise cognitively impaired should be excluded from Dignity Therapy. | No information | No information |
| Fegg et al^11^  2013  Results paper  Germany | To investigate the applicability of EBT to informal caregivers of palliative care patients and its effectiveness in with regards to psychological distress and QOL compared with a usual care. | Palliative care wards, radiation/ oncology department  life expectancy 6 months or less, one relative per patient | existential behavioural therapy versus usual care | No information | Abstract/results/discussion: No adverse events reported in the study arm that would indicate side effects of EBT. | No information | No information |
| Hudson et al^12^  2015  Australia | To evaluate a 1 to 1 psychoeducational intervention aimed at mitigating distress | Caregivers of patients with advanced cancer receiving home based palliative care | 1 to 1 psychoeducational intervention versus standard care | No information | Discussion section: An important outcome of the intervention studied here was the apparent absence of negative sequelae for caregivers in the intervention group. | No information | No information |
| Jones et al^13^  2011  Results paper  UK | To test an advance care planning discussion schedule in an exploratory patient preference trial. | oncology clinics, a hospice,  advanced cancer, no psychiatric diagnosis | Advance care planning by an independent mediator versus usual care | No information | Discussion section: advance care planning does not cause undue anxiety or depression. | No information | No information |
| Lloyd Williams et al^14^  2013  Results paper  UK | To determine if a focused narrative review could alleviate the components of suffering and anxiety and depression in advanced cancer patients | Hospice day care  Advanced metastatic disease | Focused narrative review Usual care arm offered intervention out of trial after 8 week follow up | No information | No information | Any patient found to have high scores on any measures at any time points were referred onto the hospice team and managed according to hospice practice | Any patient found to have high scores on any measures at any time points were referred onto the hospice team and managed according to hospice practice |
| McLean et al ^15^  2013  Results paper  Canada | To evaluate emotionally focused therapy on marital functioning and psychosocial outcomes among distressed couples. | Cancer centre  metastatic cancer and caregiver, distressed couples | emotionally focused therapy versus standard care | No information | Clinicians involved in both study groups monitored adherence as well as the occurrence of adverse events for the purposes of tracking and addressing any concerns. | No information | No information |
| Stein et al^16^  2013  Results paper  Australia | To determine whether an intervention could facilitate earlier DNR orders | hospital  advanced cancer , estimated life expectancy 3-12 months and caregivers | Pamphlet, discussion with a psychologist | No information | Hypothesised that the intervention was free from additional psychological harm, hypothesis supported, no evidence of worse psycho social functioning in the intervention group among patients and care givers | No information | No information |
| Volandes et al ^17^  2013  Results paper  US | To examine the effect of a video decision support tool on CPR preferences | Oncology outpatients  Advanced cancer, aware prognosis less than a year | Video Decision Support Tool for Cardiopulmonary Resuscitation and narrative versus narrative control | No information | Results section: There were no adverse events (e.g. emotional distress) in either arm of the study. | No information | No information |

1. Bernacki R, Hutchings M, Vick J, et al. Development of the Serious Illness Care Program: a randomised controlled trial of a palliative care communication intervention. *BMJ open* 2015; 5: e009032.

2. Bove DG, Overgaard D, Lomborg K, et al. Efficacy of a minimal home-based psychoeducative intervention versus usual care for managing anxiety and dyspnoea in patients with severe chronic obstructive pulmonary disease: a randomised controlled trial protocol. *BMJ Open* 2015; 5: e008031-e008031. DOI: 10.1136/bmjopen-2015-008031.

3. Houben CHM, Spruit MA, Wouters EFM, et al. A randomised controlled trial on the efficacy of advance care planning on the quality of end-of-life care and communication in patients with COPD: The research protocol. *BMJ Open* 2014; 4 (1) (no pagination).

4. Kimmel AL, Wang J, Scott RK, et al. FAmily CEntered (FACE) advance care planning: Study design and methods for a patient-centered communication and decision-making intervention for patients with HIV/AIDS and their surrogate decision-makers. *Contemporary clinical trials* 2015; 43: 172-178.

5. Kruizinga R, Scherer-Rath M, Schilderman JBAM, et al. The life in sight application study (LISA): Design of a randomized controlled trial to assess the role of an assisted structured reflection on life events and ultimate life goals to improve quality of life of cancer patients. *BMC cancer* 2013; 13 (no pagination).

6. Lo C, Hales S, Rydall A, et al. Managing Cancer And Living Meaningfully: Study protocol for a randomized controlled trial. *Trials* 2015; 16 (1) (no pagination).

7. Reid J, Scott D, Santin O, et al. Evaluation of a psychoeducational intervention for patients with advanced cancer who have cachexia and their lay carers (EPACaCC): study protocol. *Journal of advanced nursing* 2014; 70: 1174-1183. DOI: 10.1111/jan.12268.

8. Scheffold K, Philipp R, Engelmann D, et al. Efficacy of a brief manualized intervention Managing Cancer and Living Meaningfully (CALM) adapted to German cancer care settings: Study protocol for a randomized controlled trial. *BMC cancer* 2015; 15 (1) (no pagination).

9. Walczak A, Butow PN, Clayton JM, et al. Discussing prognosis and end-of-life care in the final year of life: A randomized controlled trial of a nurse-led ommunication support programme for patients and caregivers. *BMJ Open* 2014; 4 (6) (no pagination).

10. Chochinov HM, Kristjanson LJ, Breitbart W, et al. Effect of dignity therapy on distress and end-of-life experience in terminally ill patients: a randomised controlled trial. *The Lancet Oncology* 2011; 12: 753-762.

11. Fegg MJ, Brandstatter M, Kogler M, et al. Existential behavioural therapy for informal caregivers of palliative patients: A randomised controlled trial. *Psycho-oncology* 2013; 22: 2079-2086.

12. Hudson P, Trauer T, Kelly B, et al. Reducing the psychological distress of family caregivers of home based palliative care patients: Longer term effects from a randomised controlled trial. *Psycho-oncology* 2015; 24: 19-24.

13. Jones L, Harrington J, Barlow CA, et al. Advance care planning in advanced cancer: can it be achieved? An exploratory randomized patient preference trial of a care planning discussion. *Palliative & supportive care* 2011; 9: 3-13.

14. Lloyd-Williams M, Cobb M, O'Connor C, et al. A pilot randomised controlled trial to reduce suffering and emotional distress in patients with advanced cancer. *Journal of affective disorders* 2013; 148: 141-145.

15. McLean LM, Walton T, Rodin G, et al. A couple‐based intervention for patients and caregivers facing end‐stage cancer: Outcomes of a randomized controlled trial. *Psycho-oncology* 2013; 22: 28-38. DOI: 10.1002/pon.2046.

16. Stein RA, Sharpe L, Bell ML, et al. Randomized controlled trial of a structured intervention to facilitate end-of-life decision making in patients with advanced cancer. *Journal of clinical oncology : official journal of the American Society of Clinical Oncology* 2013; 31: 3403-3410.

17. Volandes AE, Paasche-Orlow MK, Mitchell SL, et al. Randomized controlled trial of a video decision support tool for cardiopulmonary resuscitation decision making in advanced cancer. *Journal Of Clinical Oncology: Official Journal Of The American Society Of Clinical Oncology* 2013; 31: 380-386. DOI: 10.1200/JCO.2012.43.9570.
